# Quantitative Analysis of Poloxamer 188 in Biotherapeutic Process Streams Using Liquid Chromatography–Triple-Quadrupole Mass Spectrometry

**DOI:** 10.1021/acsomega.3c08197

**Published:** 2024-03-22

**Authors:** Ciaran Buckley, Ciara MacHale, Jonathan Bones

**Affiliations:** † Eli Lilly Kinsale Limited, Dunderrow, Kinsale, Co. Cork P17 NY71, Ireland; ‡ School of Chemical and Bioprocess Engineering, University College Dublin, Belfield, Dublin 4 D04 V1W8, Ireland; § National Institute for Bioprocessing Research & Training, Fosters Avenue, Mount Merrion, Blackrock, Co. Dublin A94 X099, Ireland

## Abstract

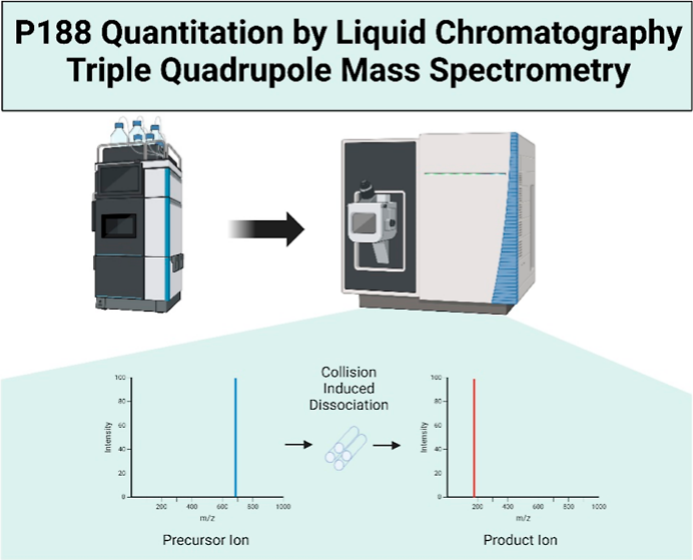

Poloxamer 188, also known as Pluronic F-68, is an excipient added to the biotherapeutic protein-manufacturing process. Poloxamer 188 (P188) is a nonionic triblock copolymer surfactant that can be used as a shear protective excipient in bioreactors. In the current study, a method for the process clearance monitoring of poloxamer 188 during downstream processing of biotherapeutics using liquid chromatography–triple-quadrupole mass spectrometry was developed and validated. Chromatographic separation of P188 was achieved using a Phenomenex, Luna 3 μm phenyl-hexyl, 150 × 2 mm column, and quantitation was achieved using a triple-quadrupole mass spectrometer operated in selected reaction monitoring mode. Linearity was assessed over a working range of 250–10,000 ng/mL. Precision and accuracy were within 15% of the theoretical spike levels assessed across the three different concentration levels. For this study, two different IgG1 antibodies were used for the method validation activities. Analyte specificity and selectivity were deemed acceptable based on no extraneous peaks present. System suitability was evaluated throughout this study in anticipation of the introduction of this method into the quality control environment. This method was successfully validated and used to monitor the clearance of poloxamer 188 in a tangential flow filtration purification step during biotherapeutic downstream processing. In addition, the capability of the method to successfully support poloxamer 188 mixing studies is presented in this work.

## 1 Introduction

Agitation is a critical process parameter in bioreactors for the large-scale production of biotherapeutics to maintain a uniform and homogeneous environment within the reactor vessel. Agitators also help to increase the rate of transfer of oxygen to the culture medium, which is essential for cell growth and metabolism. By creating a turbulent flow, agitators promote gas–liquid mixing, which enhances oxygen transfer.^1,2^ In addition to oxygen, agitators also help to enhance the mass transfer of other nutrients and metabolites within the culture medium. This can improve the efficiency of nutrient uptake by the cells, as well as facilitate the removal of waste products.^3^

However, agitators can also generate shear stress on cells, which can affect cell growth and product quality.^4−6^ Poloxamer 188 is a nonionic triblock copolymer surfactant that can be used for drug solubilization, controlled release, and for protection of microorganisms against mechanical damage.^7−9^ P188 is the most prevalent of these surfactants that is used in mammalian and insect cell cultivation due to its shear protection against sparging damage and due to it being approved by the Food and Drug Administration (FDA) as a constituent of injectable products.^10−13^ Poloxamer 188 has been shown to decrease surface tension and as a result reduce the energy expelled due to bubbles bursting within the bioreactors along with its properties to reduce the affinity of bubbles and cell attachment.^14^

**Figure 1:**
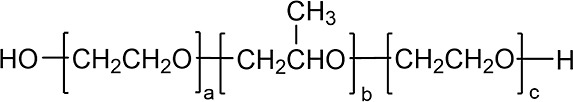
Poloxamer 188 structure.

Poloxamer 188 has an average molecular weight of 8400 Da, of which ethylene oxide makes up 80%. Poloxamer molecules are characterized by the variable values of *a*, *b*, or *c* which are visualized in Figure 1 with the average values of *a* = 75, *b* = 30, and *c* = 75.^14^ Poloxamer 188 is typically added to cell culture media at a concentration of 1–2 g/L, but this amount can vary depending on the cell line, media composition, and manufacturing process.^15^ Monitoring the clearance of poloxamer 188 is required for both process capability knowledge and to ensure that the excipient is below toxicological limits in the final process streams. Poloxamers are widely used in the pharmaceutical industry due to their previously noted properties; however, analysis of these compounds is challenging due to the compounds lacking a chromophore and complex separation behavior.^16^ The most common method used to determine the concentration of poloxamer 188 is size-exclusion chromatography with refractive index (RI) detection.^17^ RI detectors tend to have unstable baselines and lack the sensitivity required for accurately determining low concentrations of surfactants. More accurate and sensitive methods are required to ensure the removal of P188 from the process during downstream processing unit operations to ensure product safety. Takáts et al.^18^ described matrix-assisted laser desorption-mass spectrometry and electrospray ionization mass spectrometry (ESI-MS) methods for the qualitative and quantitative determination of poloxamers. This study used the average molecular weight of poloxamer 188 and a sample clean up step prior to analysis. However, the method lacked reproducibility and sensitivity, with a detection limit of 0.02% poloxamer 188.

Nair et al.^16^ described two approaches to the determination of poloxamer 188 in a water-insoluble product utilizing evaporative light scattering (ELS) and ESI-MS on a single quadrupole mass spectrometer. The ELS method determined the concentration of poloxamer 188 in solutions ranging from 0.1 to 0.3 mg/mL, and the ESI-MS method was capable of detecting as low as 25 μg/mL poloxamer 188.

In this study, a liquid chromatography–tandem mass spectrometry method (LC-MS/MS) based on selected reaction monitoring (SRM) mode analysis was developed. The method was then validated for the identification and quantitation of residual P188 in the tangential flow filtration (TFF) purification step during downstream processing. The method was validated for a quantitation range between 250 and 10,000 ng/mL P188. The method validation parameters assed were linearity, limit of quantification, precision, accuracy, and solution stability. The spiking solutions to assess precision and accuracy were all prepared at a nominal protein concentration of 10 mg/mL in an effort to streamline the method workflow. The TFF process streams were diluted to 10 mg/mL from their starting concentrations of >100 mg/mL. Two different IgG1 process streams (A and B) were used to validate the method. The method workflow allowed for a simple conversion of nanograms per milliliter of P188 to parts per million relative to the biotherapeutic protein (ng/mL P188 per mg/mL of protein).

## 2 Materials and Methods

### 2.1 Chemicals and Reagents

Poloxamer 188 was purchased from Sigma-Aldrich (Wicklow, Ireland). LCMS Optima-grade water and LCMS Optima-grade acetonitrile were purchased from Fisher Scientific (Dublin, Ireland). Formic acid was purchased from Fisher Scientific (Dublin, Ireland). Ammonium acetate was purchased from Honeywell (Dublin, Ireland). Two different IgG1 monoclonal TFF process streams, composed of different and unique matrices, were used for this study. The two process streams are termed IgG1 A and IgG1 B throughout this study. The TFF streams used for this work were supplied by Eli Lilly (Cork, Ireland).

### 2.2 Solution Preparation

Four 10,000 μg/mL P188 stock solutions were prepared by adding 2 mL of Optima-grade water (Fisher Scientific, Dublin, Ireland) into four 20 mg preweighed P188 vials (Sigma-Aldrich, Wicklow, Ireland). Each vial was then sonicated to promote dissolution. These stocks were used to prepare all working standard solutions by using a 50:50 mobile phase A and mobile phase B mix as the diluent. Two stocks were used to prepare individual sets of calibration standards, and two stocks were used to prepare quality control check standards. Each TFF process stream was diluted to 20 mg/mL in water. The 20 mg/mL TFF process streams were combined 50/50 with P188 standards to prepare the spiking solutions, resulting in solutions spiked at a nominal product protein concentration of 10 mg/mL.

### 2.3 LC-MS/MS Analysis

LC-MS/MS analysis was performed using a Thermo Scientific Vanquish Horizon UHPLC (Thermo Scientific, Germering, Germany) coupled to a Thermo Scientific Altis triple-quadrupole mass spectrometer (Thermo Scientific, San Jose, CA, USA) equipped with a heated electrospray ionization source (H-ESI source). Data acquisition and instrumentation control were performed using Thermo Scientific Chromeleon CDS, version 7.2.10. Chromatographic separations were performed using a Phenomenex Luna 3 μm phenyl-hexyl, 150 × 2 mm reversed-phase chromatography column (P/N 00F-4256-BO). Mobile phase A comprised water containing 10 mM ammonium acetate and 0.05% (v/v) formic acid. Mobile phase B comprised acetonitrile containing 10% v/v 2-propanol. LC separation parameters are provided in Table 1, briefly; the flow rate was 0.4 mL/min, with an elution gradient starting at 20% mobile phase B and increasing to 70% B over the course of 1.5 min, followed by increasing to 99% B over the course of 2 min. The gradient was held at 99% B for 1.5 min, followed by decreasing to 20% B and re-equilibrating for 1 min. The method utilizes a 20 μL injection volume. SRM analysis was used to monitor the transition from the 693 *m*/*z* precursor ion to the 177 *m*/*z* product ion. The MS source parameters used for this method are outlined in Table 2.

**Table 1:** Chromatographic Parameters

parameters	set points		
column temperature (°C)	30		
flow rate (mL/min)	0.4		
autosampler temperature (°C)	2–8		
injection volume (μL)	20		
elution profile	time (min)	% MPA	% MPB
	0.0	80	20
	0.9	80	20
	1.5	30	70
	3.5	1	99
	5.0	1	99
	5.5	80	20
	6.5	80	20

**Table 2:** MS Source, Instrument, and SRM Method Parameters

parameters	set points
mode	SRM
SRM transition	693–177 *m*/*z*
polarity	positive
positive-ion voltage (V)	3500
sheath gas	50
aux gas	10
sweep gas	0
Ion-transfer tube temperature (°C)	325
vaporizer temperature (°C)	350
precursor (*m*/*z*)	693.45
product ion 1 (*m*/*z*)	177.08
collision energy	22.7
RF lens	68
min dwell time (ms)	399.27
Q1 resolution	0.7
Q3 resolution	1.2
CID gas (mTorr)	1.5
source fragmentation (V)	10

## 3 Results and Discussion

### 3.1 LC-MS Optimization

**Figure 2:**
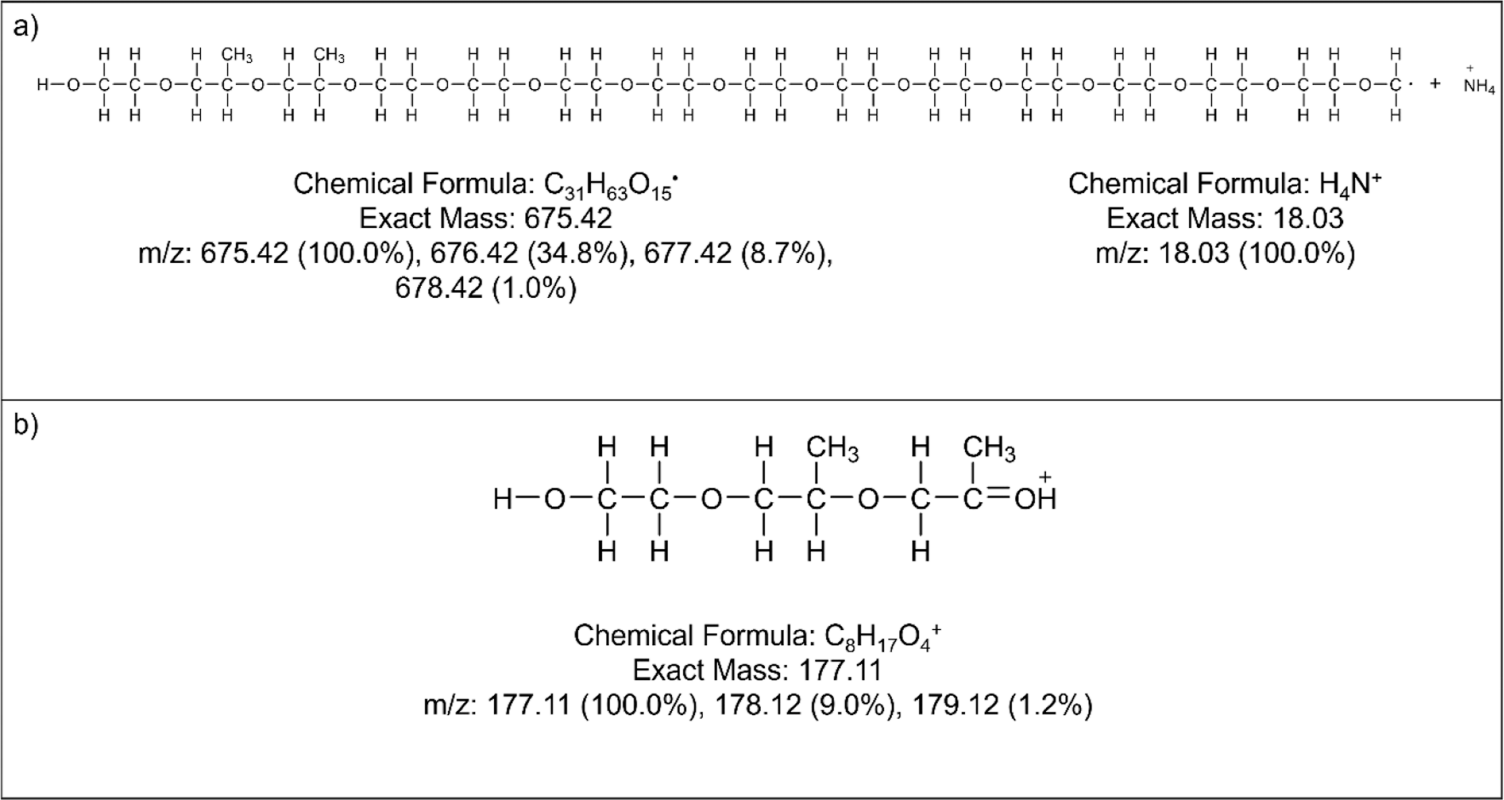
(a) Proposed structure and adduct formation for the 693 precursor ion and (b) proposed structure for the 177 product ion.

**Figure 3:**
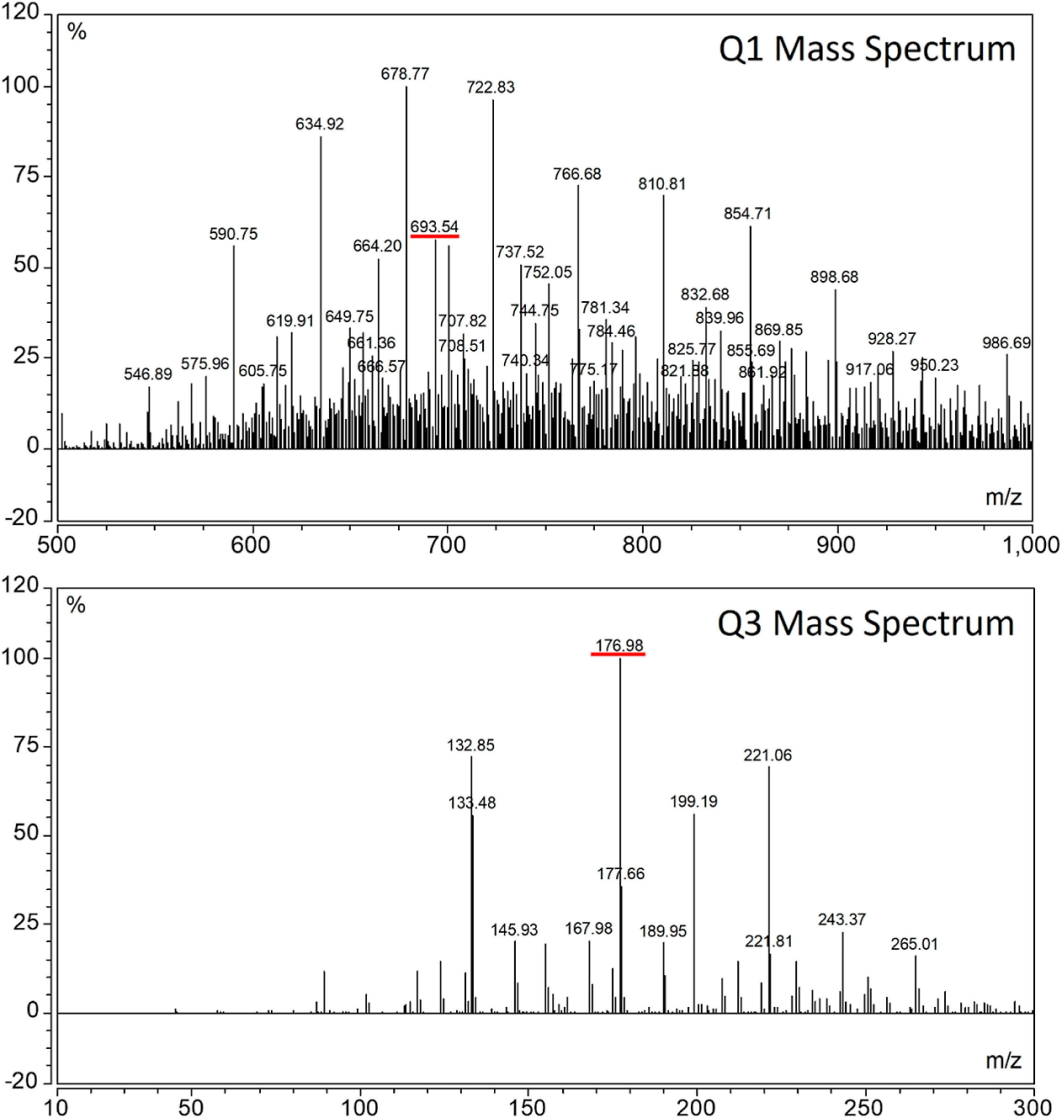
Q1 mass spectrum for the 693 precursor ion and Q3 mass spectrum for the 177 product ion.

To identify a stable and reproduceable SRM transition, 1 μg/mL P188 stock was infused into MS using a syringe pump at 100 μL/min with the instrument set to scan. Source fragmentation was ramped from 0 to 30 V to identify a stable precursor ion. A stable precursor ion of 693 *m*/*z* was identified during the initial feasibility studies, which potentially corresponds to the ammonium adduct of C_31_H_63_O_15_*. No stable adducts were observed when P188 was infused in negative mode, and as such, positive-ion mode was chosen to proceed with further development work. Using product ion scan mode, the collision energy was ramped from 0 to 100 V and CID gas was ramped from 0 to 3 mTorr. Argon was used as the collision gas for this study. During this experiment, a selection of product ions at low abundance was performed, but a stable and reproducible 177.08 *m*/*z* product ion was identified, which possibly corresponds to C_8_H_17_O_4_^+^. The proposed precursor and product ion structures are visualized in Figure 2. The precursor and product ion mass spectra are listed in Figure 3.

Upon the identification of a stable SRM transition that provided the sensitivity required, columns and the mobile phase for chromatographic analysis of P188 were evaluated. The column chosen for this study was a Phenomenex Luna 3 μm phenyl–hexyl, 150 × 2 mm due to the high surface area, which offered superior chromatography to that of more standard C18 stationary phases. During the development of the method, it was identified that depending on the lots of stationary phases used, the polyoxyethylene oxide hydrophilic chains of P188 separated on column resulting in varying peak resolution. To resolve this, 10% 2-propanol was added to mobile phase B to improve the peak retention. In addition, an MS smoothing factor of 21 was applied postacquisition to ensure that if splitting of the main peak occurred, all forms of P188 would be quantitated, which was the main goal of the method development.

Using the chromatographic conditions and MS settings developed, P188 was detectable and quantitated in the presence of therapeutic proteins in bioprocess streams at a retention time of 3 min in a 6.5 min assay.

### 3.2 Method Validation

Method validation activities were performed following the ICH Q2R1 method validation guidelines. Specificity was demonstrated by spiking both of the IgG1 process streams with a nominal concentration of 1000 ng/mL P188 and comparing the chromatograms to a diluent blank and unspiked process stream. The method quantifies the amount of P188 in a sample through targeted MS/MS reaction monitoring, in which a specific precursor ion is isolated and fragmented to form a specific product ion. This product ion peak area is quantitated, which yields a reportable result. This MS/MS transition is specific to P188 under the method conditions used in this study. Repeatability was evaluated by spiking 1000 ng/mL P188 into two IgG1 TFF process streams and quantitating the level of P188 present. The process streams were analyzed prior to spiking to calculate the amount of P188 present to enable calculation of the recovery of the 1000 ng/mL P188 spike. Percentage recovery was calculated by expressing the measured P188 amount vs the theoretical amount spiked. Six individual spiked solutions were prepared, and one injection of each solution was performed. Accuracy was evaluated by spiking the two process streams at three different concentration levels. All solutions were quantitated on a freshly prepared P188 calibration curve. The intent of the study was to spike at a low, medium, and high level of P188 that would cover the concentration range reflective of the potential production values. Samples were spiked with 1000, 2500, and 5000 ng/mL P188. The process streams were spiked at a nominal protein concentration of ∼10 mg/mL which equates to a final concentration of 100, 250, and 500 ppm (ng P188/mg protein). Unspiked solutions of the process streams were analyzed and yielded results less than the limit of quantitation (LOQ), and hence, no concentration correction was necessary for the levels of P188 present prior to spiking. The linearity of the method was evaluated for P188 between a concentration range of 250–10,000 ng/mL in diluent. This equates to a range of 25–1000 ppm (ng P188/mg protein) when samples are prepared at a protein concentration of 10 mg/mL. Two separate preparations of this calibration curve were prepared containing six individual calibration levels. Linearity was deemed acceptable if the coefficients of determination (*R*^2^) for both calibration curves were greater than or equal to a value of 0.99 for P188. Additionally, the calculated concentration of each calibration standard was required to be within ±25% of the theoretical concentration. A check standard was also prepared separately to ensure an acceptable method performance. The LOQ was evaluated through serial dilution of P188 until a stable, repeatable peak was measurable. The signal-to-noise ratio was then calculated for the peak. Solution stability was evaluated for both standard and diluted sample solutions for each of the IgG1 process streams. Standard stability was evaluated by comparing the result for a freshly prepared check standard solution to that of the same standard stored at 2–8 °C for 3 days. The check standard was quantitated using a freshly prepared calibration curve on both days. Sample stability was evaluated by assessing one preparation of each of the accuracy samples; 1000, 2500, and 5000 ng/mL P188 spiked samples stored at 2–8 °C for 3 days. The levels of P188 in these spiked samples were quantified by using a freshly prepared calibration curve on both days.

#### 3.2.1 Linearity Assessment

Two individual P188 calibration curves were prepared. This method utilizes a quadratic regression calibration curve, *y* = *ax*^2^ + *bx* + *c*. Table 3 summarizes the results of the regression analysis of the method. Both calibration curves produced an acceptable coefficient of determination (*R*^2^) results of greater than or equal to a value of ≥0.99. The calculated concentrations of each calibration standard were within ±25% of the theoretical concentration and are presented in Tables 4 and 5.

**Table 3:** Linearity Regression Data

parameter	linearity setup 1	linearity setup 2
nominal range (ng/mL)	250–10,000	250–10,000
coefficient of determination (*R*^2^)	1.0000	1.0000
*y*-intercept (C0)	133.616	–835.992
slope (C1)	14.254	15.234
curve (C2)	–4.195 × 10^–4^	–4.230 × 10^–5^
residual sum of squares	12867.80	13186.57

**Table 4:** Linearity Data for Calibration Curve Setup 1

standard	theoretical concentration (ng/mL)	calculated concentration (ng/mL)	absolute difference (ng/mL)	% recovery
calibration standard 1	250.00	246.95	–3.05	98.8
calibration standard 2	500.00	499.03	–0.97	99.8
calibration standard 3	1000.00	1000.94	0.94	100.1
calibration standard 4	2000.00	2006.97	6.97	100.3
calibration standard 5	5000.00	4994.64	–5.36	99.9
calibration standard 6	10000.00	10001.69	1.69	100.0

**Table 5:** Linearity Data for Calibration Curve Setup 2

standard	theoretical concentration (ng/mL)	calculated concentration (ng/mL)	absolute difference (ng/mL)	% recovery
calibration standard 1	250.00	245.87	–4.13	98.3
calibration standard 2	500.00	502.31	2.31	100.5
calibration standard 3	1000.00	1005.12	5.12	100.5
calibration standard 4	2000.00	1996.17	–3.83	99.8
calibration standard 5	5000.00	5000.57	0.57	100.0
calibration standard 6	10000.00	9999.96	–0.04	100.0

#### 3.2.2 Specificity and Sensitivity Assessment

**Figure 4:**
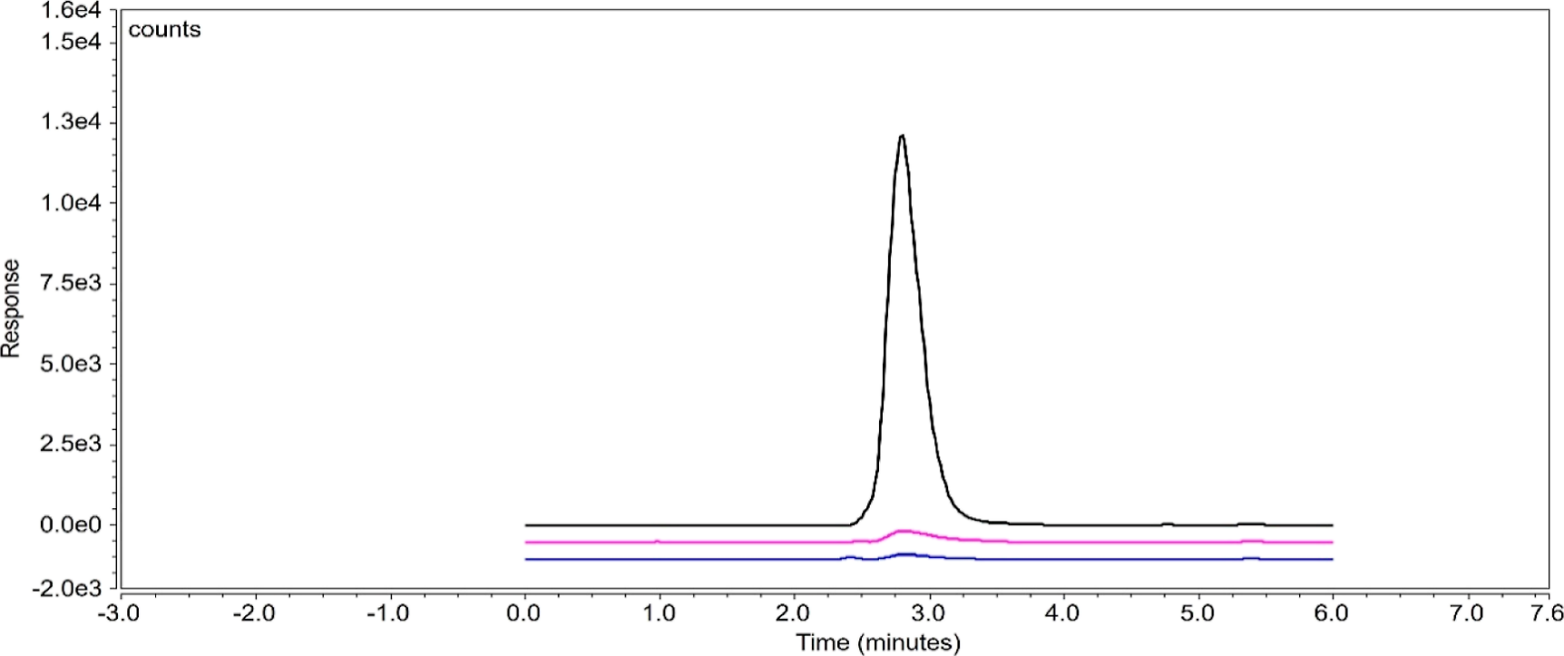
Overlay of diluent blank (blue), an unspiked TFF process stream (pink), and 250 ng/mL calibration standard (black).

Specificity was demonstrated through the preparation of blank, unspiked process streams and a set of calibration standards. The unspiked process streams and blanks showed no significant peaks for the P188 transition. A peak was observed in each calibration standard, but it was not present in the specificity controls. To determine the LOQ of the method, P188 standards were prepared at incremental concentrations. Standards were injected with higher concentrations of P188 until a stable, repeatable peak was observed. This corresponded to a 250 ng/mL standard of P188, which equates to 25 ppm (ng P188/mg protein) when samples are prepared at a concentration of 10 mg/mL. A signal-to-noise ratio of 116.6 was calculated for the 250 ng/mL standard. The purpose of this study was to design a quantitative method, and as a result, the limit of detection was not evaluated. Any results below the 250 ng/mL calibration standard are reported as below the LOQ of the method. Figure 4 illustrates an overlay of a blank, an unspiked TFF process stream, and a 250 ng/mL calibration standard.

#### 3.2.3 Accuracy and Repeatability

Intraday accuracy data pertaining to the analysis of both IgG1 process streams are detailed in Table 6. The mean accuracy recovery % values at each spiked level (low, medium, and high) were 99.7, 101.3, and 101.5%, respectively, for the IgG1 (A) molecule. The mean accuracy recovery % values for the IgG1 (B) molecule were 90.6, 94.4, and 103.2%, respectively. The method demonstrates acceptable precision at each of the three spike levels for both IgG1’s. Repeatability was assessed for both molecules at a single concentration of 1000 ng/mL P188. Acceptable repeatability results of CV % values of 1.4 and 2.2% for the IgG1 (A) and IgG1 (B), respectively, were obtained and are displayed in Table 7.

**Table 6:** Accuracy Data^a^

		accuracy			
	spike (ng/mL)	recovery (%)	CV (%)	95% LCL	95% UCL
IgG1 (A)	1000	99.7	0.3	99.0	100.4
	2500	101.3	0.5	100.2	102.4
	5000	101.5	1.0	99.0	104.0
IgG1 (B)	1000	90.6	2.4	85.3	95.9
	2500	94.4	1.7	90.4	98.3
	5000	103.2	1.0	100.6	105.8

a CV: coefficient of variation (*n* = 3). LCL: lower confidence level. UCL: higher confidence level.

**Table 7:** Precision Data^a^

		precision			
	spike (ng/mL)	recovery (%)	CV (%)	95% LCL	95% UCL
IgG1 (A)	1000	99.6	1.4	98.1	101.1
IgG1 (B)	1000	89.6	2.2	87.5	91.7

a CV: coefficient of variation (*n* = 6). LCL: lower confidence level. UCL: higher confidence level.

#### 3.2.4 Solution Stability Assessment

Analytical solution stability was evaluated for the calibration check standard and spiked TFF process streams for IgG1 A, and the data are detailed in Table 8. The solutions were stored for 3 days at 2–8 °C and quantitated via freshly prepared calibration standards. The aged/fresh ratio of the check standard was 100.9%. The aged/fresh ratios of the 1000, 2500, and 5000 ng/mL P188 spiked TFF samples were 98.0, 100.7, and 100.4%, respectively. Based on this data, both the check standard and spiked TFF samples are stable for up to 3 days stored at 2–8 °C.

**Table 8:** Stability Assessment of Standard Solutions and Spiked Samples

T3 days	T3 days concentration (ng/mL)	initial concentration (ng/mL)	aged/fresh ratio (%)
1000 ng/mL	978.5	998.7	98.0
2500 ng/mL	2536.4	2519.7	100.7
5000 ng/mL	5099.8	5079.3	100.4
check standard	1022.1	1013.4	100.9

#### 3.2.5 Determination of System Suitability

System suitability data was gathered and evaluated during this study to ensure that the method was capable of being introduced into a quality control laboratory for routine use. Parameters evaluated consisted of the following: (a) linearity assessment using standards from ∼250 to 10,000 ng/mL; (b) determination of the presence of interfering peaks, e.g., carryover response; (c) and performance checking throughout the run using a check standard post calibration curve. Carryover and check standard system suitability parameters are displayed in Tables 9 and 10, respectively.

**Table 9:** Carryover System Suitability Assessment

carryover parameters	setup 1	setup 2
blank area	99	76
250 ng/mL standard area	3628	3583
% of lowest standard	2.7	2.1

**Table 10:** Check Standard System Suitability Assessment

check standard parameters	setup 1	setup 2
area	14,147	15,005
calculated amount (ng/mL)	1013	1060
theoretical recovery %	101.3	106.0

#### 3.2.6 Method Applications

**Figure 5:**
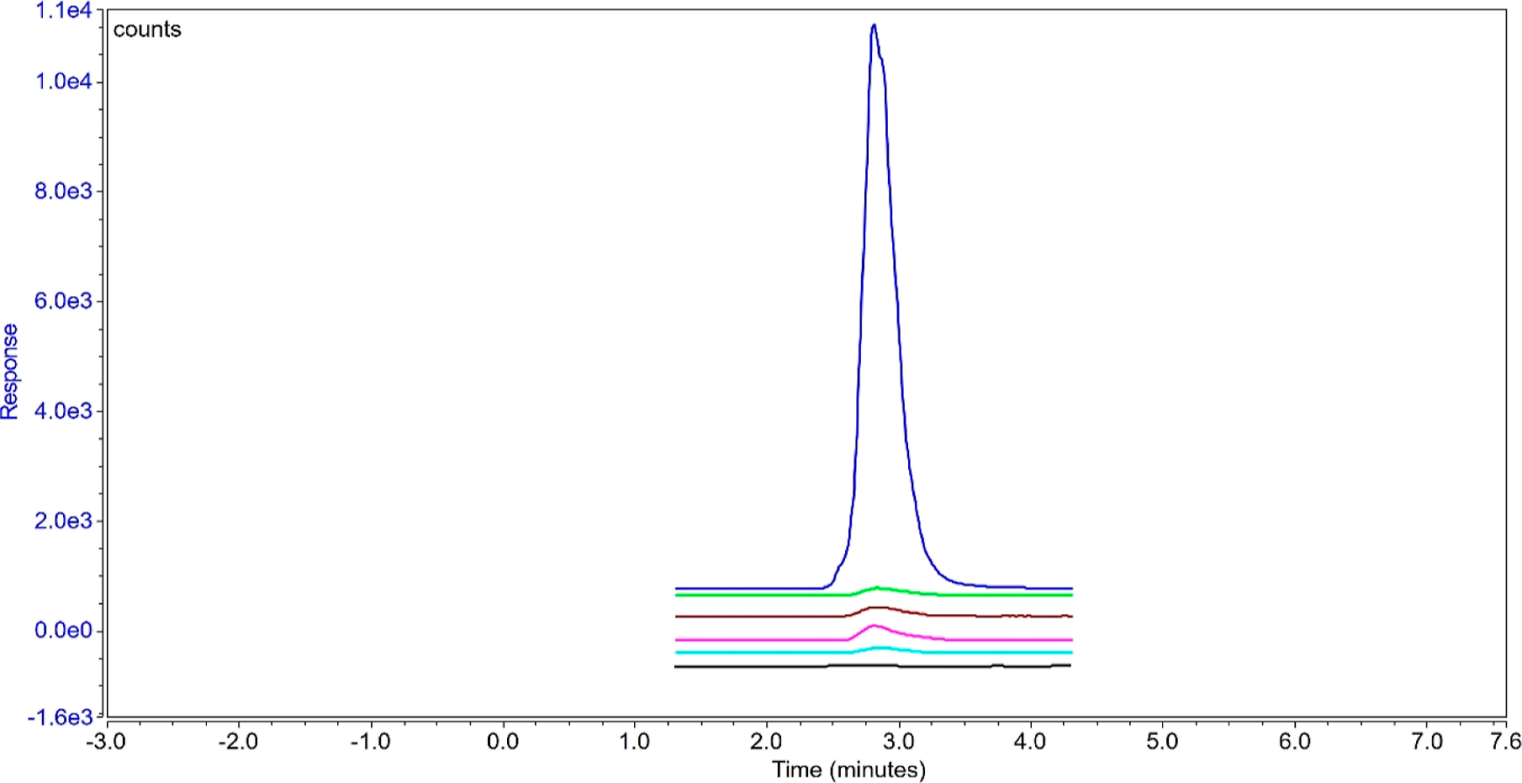
Overlay of diluent blank (black), TFF sample 1 (light blue), TFF sample 2 (pink), TFF sample 3 (brown), TFF sample 4 (green), and 250 ng/mL calibration standard/LOQ standard (blue).

Detailed in this section are two examples of analytical method applications. First, the method was used for its originally designed application in which TFF unit operation samples were analyzed for residual poloxamer 188. Four batches of development TFF were analyzed and yielded results less than the LOQ. The chromatograms of the samples analyzed are displayed in Figure 5.

The second application of this method was to support a poloxamer 188 mixing study. A small-scale mixing study was executed to determine when P188 is added to an aqueous solution and mixed and at what time point would the solution become homogeneous. The samples for the mixing study were provided for analysis directly from the mixing vessel. For the study to be deemed acceptable, a sample should yield a result of 0.04% w/v P188 ± 10% at a specific time point, and all further time points should remain constant at this level of P188. The scope of this work was to use the method developed to determine the concentration of poloxamer 188 in aqueous-based samples at specific time points. The target concentration of the mixing study was 0.04% w/v P188 which corresponds to 400,000 ng/mL. Due to the elevated levels of Poloxamer 188 anticipated to be present in the samples and the working range of the analytical method, a 1 in 100 gravimetric dilution was applied to each sample prior to analysis. The results obtained were corrected for the gravimetric preparation and converted to % w/v. The mixing study was deemed successful based on the analytical data successfully meeting the acceptance criteria. The data is presented in Table 11.

**Table 11:** Poloxamer 188 Mixing Study Results

sample time point	corrected concentration (% w/v)
T0	none detected
T20	0.039
T25	0.039
T30	0.038
T35	0.040
T55	0.039
T75	0.039
T95	0.039
T105	0.040

## 4 Discussion and Conclusions

In this work, a method for the identification and quantitation of poloxamer 188 in biotherapeutic process streams is presented. Using two different IgG1 antibodies, the method was developed and validated demonstrating greater sensitivity than what has been previously presented in the literature.^16^ The data detailed in this study demonstrate the accuracy and precision of the method at low-level trace analysis. With a requirement for minimal sample preparation and a 6.5 min run time, the method presented is an analytical solution for the rapid monitoring of P188 clearance in bioprocessing purification unit operations. In addition to the methods intended use, the method was also applied to facilitate a poloxamer 188 mixing study as detailed in Section 3.2.6. The accuracy and precision of the results demonstrate the method’s robustness and selectivity at quantitating poloxamer 188. The method presented is a validated and reliable analytical tool that can be used in the pharmaceutical industry to support process validation and additionally can be introduced into a cGMP laboratory as a quality control assay.
